# Cognitive neurosciences: A new paradigm in management and outcome of schizophrenia

**DOI:** 10.4103/0019-5545.64575

**Published:** 2010

**Authors:** Amresh K Shrivastava, Megan E Johnston

**Affiliations:** University of Western Ontario, Lawson Health Research Institute, London, Consultant psychiatrist and physician-team leader, Early Psychosis Program, Regional Mental Health Care, 467 Sunset Drive, St.Thomas, N5P 3V9, Canada; 1 University of Toronto, Department of Psychology, 100 St. George Street, Toronto, ON M5S 3G3, Canada

Cognitive neuroscience of schizophrenia has truly emerged as a connection between neurobiological changes and psychological processes. The current state of research is reflective of the evidence that brain changes correlate with psychological dysfunctions and may have a causal relationship. Thus, a significant number of behavioral traits, functional limitations and psychological vulnerabilities can now be explained on the basis of underlying neurobiological deficits. Cognitive neurosciences have thus emerged as one of the ‘missing-links’ in understanding the phenomenology of schizophrenia.

Schizophrenia is an illness with functional disability as a major issue.[1] Outcome in schizophrenia has always been a complex matter, as the concept of outcome changes not only with scientific developments but also with rising consumer expectations. In the past, measures of outcome have focused only on clinical symptoms, but today it is recognized that social outcomes are an equally important consideration. In order to best capture the quality of life of individuals affected by schizophrenia, it has become necessary to consider social, occupational, and cognitive variables while defining outcome. Re-appearance of cognitive functions in the scene of schizophrenia outcome is viewed as a turning point in the research progress. It has provided various arguments as well as mechanisms to look beyond the symptom clusters. Cognitive functions have become a visible link between dysfunction, disability, symptomatology and neurobiological changes.

The issue amongst clinicians is whether it will become a measurable marker for clinical outcomes, whether therapeutic interventions can change cognition functions, and whether cognitive enhancers will become the central therapeutic focus in future. Some of these issues have the potential to change the focus from treatment to prevention in psychiatrists’ practice. The implications are much broader for clinical practice and the most significant question is whether cognitive enhancers effective in schizophrenia will also be useful in treating the ‘risk-syndromes’. This paper deals with some of the basic findings of cognitive research, in relation to outcome in schizophrenia and atypical antipsychotics.

Outcome status for individuals with schizophrenia over a 100-year span, from 1895 to 1985, in a meta-analysis of published literature, shows good outcome limited to about 30% of individuals before the mid nineties and less than 37% of individuals in the late nineties. It seems clear that patients were, and are, continuing to suffer from dysfunction and disability.[2]

Outcome in schizophrenia is multidimensional, and has typically been heterogeneous and variable across cultures and regions. Cognitive function has recently emerged as an independent domain of psychopathology in schizophrenia. Its re-emergence and new significance is obvious from the proposal that cognition needs to be a diagnostic criterion for schizophrenia in the forthcoming Diagnostic and Statistical Manual of Mental Disorders (DSM V).

The cognitive functions are also significantly correlated with the level of functioning. It is expected that effective treatment in schizophrenia will significantly enhance cognitive function and thereby result in good level of functioning. Atypical antipsychotics are the mainstream treatment for schizophrenia, which reduce symptoms significantly, in three psychopathological domains: positive symptoms, negative symptoms and general psychopathology. There is an expectation that these molecules would also significantly enhance cognitive function.

Considering the emerging findings in the literature, it is important to consider measures to enhance cognitive functions or at least prevent progressive cognitive impairment in schizophrenia. We have seen an increasing emphasis on the importance of cognition in understanding psychosis over the past 20 years. There is ample evidence that a large percentage of individuals with schizophrenia suffer from impairment in a range of cognitive domains, and there is growing evidence that the level of cognitive impairment predicts functional abilities in schizophrenia (social, occupational, and living status). The hope now is that by improving cognitive function among individuals with schizophrenia, we may be able to improve functional outcome in this very debilitating illness and thus improve the quality of life for individuals with schizophrenia and address important public health and humanitarian concerns.

The field of cognition has re-affirmed Kraepelanian thought. Today there is a clearer understanding of etiopathogenesis of schizophrenia, outlining changes in the timeline from vulnerability to symptom onset explaining role of genetics, neurobiology, social and psychological stressors.[3] One of the fundamental deficits based upon neurodevelopmental theory has been the presence of cognition. Despite good evidence there is lack of clarity and several important questions to understand the complex issue of cognition and psychosis are unanswered e.g. Should cognitive function be included as one of the diagnostic criteria for schizophrenia? Is it important to include assessment of cognition in DSM-V? How should we measure cognition? Should cognition be assessed for all psychotic disorders?[4]

Cognitive impairment is present at the onset and throughout the course of schizophrenia. Research has found that cognitive deficits correlate well with clinical symptoms, and both the severity and duration of untreated illness impact the level of functioning.[5] Deficits across neurocognitive domains such as attention, working memory, language skills, and executive functioning tend to be moderate, with the most pronounced deficits found in verbal learning and memory. All these neurocognitive domains are related to adaptive and social skills, with executive functions and verbal learning and memory showing more variance across more domains than other neurocognitive variables. Negative symptoms and neurocognitive domains, although correlated, are distinct and have differential pathways of change with treatment. General psychopathology symptoms, such as depression and anxiety, may become important treatment targets as strategies are developed for translating cognitive enhancement to real-world functional performance.[6] Cognitive functions are possibly the best predictors of adaptive outcome.

Presently, various data tend to assert that cognitive dysfunctions are the core disturbance in schizophrenia and their severity is predictive of the course of the disease. It is also clear that the disturbances measured in cognitive tests are neither the consequences of positive or negative symptoms, nor related to motivation or global intellectual deficit, or to anti-psychotic medication. It is also known that the severity of cognitive symptoms is a better indicator of social and functional outcome than the severity of the negative or positive symptoms. The patients who have the most severe cognitive deficits during the first episode of the disease are most likely to present a chronic and severe form later on.[7] There is also support for longitudinal correlation between cognitive ability at base and subsequent functional outcome.[8] Studies have further demonstrated the predictive power of cognitive status for outcome, level of functioning of these patients and their quality of lives. Cognitive factors are able to predict ability to sustain employment, daily living activities and overall quality of life.[9] These reports have been replicated again and again and have provided a new dimension to schizophrenia research.[10] Real-world adaptive life skills are predicted by neuropsychological performance, symptoms, and functional capacity. The correlation of cognition and outcome is a complex. Neuropsychological performance contributes little to the prediction of real-world performance after accounting for functional capacity. In some domains, negative and depressive symptoms influenced real-world performance while not relating to functional capacity or neuropsychological performance.[11] The aspects of cognition that are specifically impaired in schizophrenia are verbal memory, working memory, motor function, attention, executive functions, and verbal fluency. Cognitive disturbances are thus very important in several fields of research in schizophrenia such as: understanding the psychopathology, epidemiology (indicators of vulnerability), genetics (endophenotypes), neuroimaging (including functional neuroimaging), and psychopharmacology (they can be used as a parameter of evaluation in therapeutic trials with new molecules, or cognitive psychotherapy). Some areas of cognitive functioning are not affected by schizophrenia. However, the most severe deficits are seen in executive functioning, attention and memory. Of these, memory in its various constructs is the most directly linked to cholinergic function. Researchers are now looking at the possibility of reversibility and prevention in the realm of cognitive dysfunction.[12]

One of the difficulties has been in understanding the pathways from cognitive dysfunction to symptoms and functional recovery, some attempt has been made to address the issue of a viable ‘model’. Social cognition has been suggested to be an important mediating variable in the relationship between neurocognition and functional outcome. The study by Bell *et al*. highlighted the direct effects of neurocognition on rehabilitation outcome and indirect effects mediated by social cognition and social discomfort. This model, they claim, is a good fit to the data and far superior to another model where only social cognition was the mediating variable between neurocognition and rehabilitation outcome. Neurocognition affects social cognition and poorer social cognition leads to social discomfort on the job, which in turns leads to poorer rehabilitation outcomes.[13]

Advancement in cognitive neuroscience has been a very significant contributor to new understandings of outcome in schizophrenia.[14] Recently, there has been a re-emergence of cognitive factors as critical indicators of outcome, after their significance was indicated by several key studies appearing in last twenty-five years.[15] While historically the position of cognition in schizophrenia has waxed and waned, it is now understood that cognitive impairment is a core and independent domain in schizophrenia.[16,17]

Cognitive dysfunction involves both structural and physiological brain impairment and, thus, drugs that can bring about improvements in cognitive functioning stand to make vast advances in the quality of lives of individuals affected by schizophrenia.[18] This is particularly critical in light of the fact that one cross-sectional study of individuals with chronic schizophrenia found that 80% subjects showed cognitive dysfunction.[19]

Cognitive dysfunction has been found to have several neuroanatomical correlates e.g. thalamus,[20] prefrontal cortex, and limbic – hippocampus regions. Studies have also given some support to regional brain dysfunction in cognition in schizophrenia (i) fronto-temporal-limbic dysfunction; (ii) abnormal connectivity or ‘miswiring’; (iii) aberrant neurodevelopment; and (iv) neurodegeneration and neural injury.

The challenge for research is to identify the molecular pathways where abnormalities culminate in the highly diverse features of the disorder. Researchers argue that candidate pathways must be able to account for the developmental and deteriorative clinical profiles and the global and focal neuropsychological deficits, as well as the various patho-anatomical abnormalities that indicate aberrant cytoarchitecture and connectivity in the absence of neurodegeneration or other obvious evidence of post maturational neural injury.[21]

Phenomenological cognitive dysfunction and/or deficit are observed across all phases of psychosis. It is not only present in frank psychotic episode but also during prodromal phase and ultra-high risk candidates. It has also been demonstrated to be present in first-degree relatives of schizophrenia.

Impairment of social cognition is another exciting research, which has attempted to correlate emotional recognition to social cognition. Such data suggests that impairments in social cognition may be unique endophenotypes for schizophrenia.[22]

There is ongoing investigation into genetic vulnerability that unfolds into abnormalities of neurotransmitter systems and dysfunctions related to various sub-types for cognition. Another interesting development has been determination of neurochemical correlates of cognitive dysfunction, which form the arguments for pharmacological intervention for cognitive domains. The success in this area has been under scrutiny recently.

One of the most difficult questions in cognition research has been the issue of measurement. A number of instruments have been developed and tried in clinical research. From the perspective of standard of care and clinical feasibility we need neuropsychological tests, which are easy to administer in clinical settings, remain reflective of current scientific consensus and possess high reliability, sensitivity and specificity.

There are ongoing efforts in this direction. The project of National Institute of Mental Health, USA, has played a significant role in bringing this issue to the forefront. Its initiative “New Approaches to Assessing and Improving Cognition in Schizophrenia” for “Measurement and Treatment Research to Improve Cognition in Schizophrenia” or MATRICS is expected to provide tangible direction on the issue of how to measure cognitive functions effectively and also how to improve upon cognitive dysfunction. We hope that this report will help researchers, industry, and funding agencies meet the urgent need for novel pharmacotherapeutics for cognitive impairment in schizophrenia and useful neurobiological indexes to track the resulting improvement in cognitive function.[23] Optimizing the application of cognitive neuroscience to new drug development will require a major commitment by multiple investigators to task development and a thorough psychometric evaluation of both behavioral and neuroimaging measures.

The new focus on cognitive factors and the subsequent new data has led healthcare workers to believe that the outcome for patients with schizophrenia can be changed and possibly enhanced by improving cognition.[24] Additionally, research in neurocognition has begun to reveal biological underpinnings that contribute to understanding the underlying psychopathology of schizophrenia and issues related to course, outcome and treatment strategies.[25] Accordingly, this has led to investigations into possible strategies for enhancing cognition.

Currently, there are two strategies in this regard. The first, cognitive preservation and prevention, is based in the enthusiastic movement of early psychosis, first episode psychosis, and the current focus of research on ultra high-risk candidates. This strategy attempts to prevent cognitive decline through early intervention, thereby limiting disability.[8] Prevention of cognitive decline and the maximization of functional and social outcome has been the core philosophy behind the early psychosis service program development. The second strategy involves cognitive enhancement through: a) pharmacological methods, or b) non-drug therapies, the most successful amongst these are cognitive behavioral approach for cognitive remediation.

New approaches to the measurement of cognition in schizophrenia include the use of tasks from experimental cognitive psychology to examine the integrity of specific cognitive systems and the application of these tasks in noninvasive neuroimaging (e.g., functional magnetic resonance imaging [FMRI]) studies that directly measure the effects of drugs on cognition-related brain activity. These approaches offer many advantages, including the isolation of specific cognitive systems that may be conserved across species. These developments have the potential to transform the early human phases of drug development and streamline decision making at this critical point in the process. In terms of using pharmacological methods to enhance cognition, the atypical antipsychotics have a unique position. Atypical antipsychotics have been found to increase cognition in schizophrenia, with multiple studies confirming this enhancement.[9]

In an analysis of 20 published studies,[26] significant changes have been effectively demonstrated for each cognitive domain (verbal fluency, secondary memory, vigilance, visuomotor skills, spatial function, executive function, immediate memory). This analysis examines the magnitude shown by the atypical antipsychotics in their ability to improve cognition, and included all second-generation antipsychotics (e.g. risperidone, olanzapine). Verbal fluency, secondary memory (very fortunately because of its influence on social and work function), and attention all demonstrated moderate effect sizes in their ability to improve cognitive skills. In general, the atypical antipsychotics provide about 40% of the variation in basal levels. Executive function and working memory in the study were reported to have shown little overall effects in response to the atypical antipsychotics.[26] Thus, the new antipsychotics provide an initial step toward restoring cognitive deficits, and we do believe that it is a functionally significant effect.

In contrast to present views, in earlier days when cognitive decline was initially established in schizophrenia, the conventional antipsychotics used at the time were blamed for this deficit. There was almost near consensus in the scientific community that the antipsychotics were responsible for this decline. Extra pyramidal symptoms were also used to explain this deficit. The scenario began to change with introduction of clozapine, the first second-generation antipsychotic,[12] and particularly in response to two important findings that came out of research by Herb Meltzer and his team regarding clozapine;[12,27] that clozapine can enhance cognition, and that the improvement in quality of life of clozapine patients, who were chronic in nature, is possibly related to its effect on cognitive domain and negative symptoms.

There was sufficient euphoria in the community regarding chronic patients showing improvement and it seemed like a second revolution after the invention of chlorpromazine. Unfortunately, after the initial excitement surrounding second-generation antipsychotics, more recent and more rigorous trials have dampened this enthusiasm. All second-generation antipsychotics introduced after clozapine were explored for their ability to enhance cognitive skills with only equivocal results and no clear message. Various studies demonstrated that risperidone, olanzapine, and quetiapine all enhance cognition to an appreciable state.[28] Some studies showed more favorable results for olanzapine and others for quetiapine.[29] It was even argued that atypical antipsychotics, unlike conventional antipsychotics, enhanced cognition by working on muscarnic receptors in some degree. Further, they do so by increasing nerve growth factors, by preventing progressive brain tissue loss associated with psychosis and by stimulating neurite extension, neurogenesis and cell survival.[30]

This has been the position until very recently. Unfortunately, all the trials of atypical antipsychotics had some serious methodological issues, most were short-term studies on small numbers of patients and each used different types of batteries. The results were variable, with some research finding haloperidol to enhance cognition while others found no effect. The effect sizes of the changes seen in cognitive scores were very small, indicating little enhancement;[28] however, the belief in the cognitive enhancement effects of atypical antipsychotics survived. The cognitive effects of atypical antipsychotics were explained with theories of neuronal plasticity and neuroreceptors. Through animal studies in addition to studies in humans (chronic users of antipsychotics and drug naïve patients) it was found that atypical antipsychoics enhance nerve growth factor and provide neuroprotection.[30] One major advantage of atypical antipsychotics is the discovery of the mechanism of action on the receptor system, which includes those receptors found to be important in the neurobiology of cognition.[31] This neurobiological merit and ‘superiority’ coupled with significant change in brain-derived neurotropic factor (BDNF) provided an explanation for mild to modest effects in favour of atypical antipsychotics in improving cognition.

In contrast to the view that atypical antipsychotics enhance the outcome and improve functional ability in patients suffering from schizophrenia, recent studies have cast doubt on this property of atypical antipsychotics. Although few major studies published in last 3-4 years changed this perception, the Clinical Antipsychotic Trials of Intervention Effectiveness (CATIE) study[22] developed a comprehensive neuropsychological battery where scores were converted to be compared easily. This study measured cognition in risperidone, olanzapine, quetiapine, ziprasidone and perphenazine at two months, six months and 18 months in large number of patients in two different phases. This study concluded that perphenazine provided patients with maximum cognitive benefit; however, cognitive benefits provided by atypical antipsychotics were marginal. Some benefit was seen at two months and was sustained though 18 months, but no additional benefits were seen at 18 months.[32]

Further research by Keefe and colleagues[33] compared quetiapine, olanzapine and risperidone at 12 weeks and 52 weeks and concluded that the benefit was not significant. Michael Green, in an editorial for the American Journal of Psychiatry in 2007, wrote that ‘*hope from atypicals has been damaged and for cognitive enhancement it appears that we have to look somewhere else*”.[34]

Thus, the relationship between cognition and atypical antipsychotics has come full circle and we are now in the phase of finding newer targets for cognitive enhancement. This is a matter of concern. Though the findings are new, it is difficult to understand from the research designs and the details available about these studies as to why this information was not reflected in earlier studies. The new research leaves clinicians disappointed. It is not possible to easily brush aside these new findings, as these results come from research undertaken very carefully with conscious control of every aspect of design.

Importantly, the role of atypical antipsychotics in cognitive enhancement should not be fully abandoned, as Sumiyoshi[35] points out the fact that the paradoxical effects of antipsychotics on cognition may be attributable to duration of treatment, dose, and the type of cognitive ability. For example, treatment with risperidone in early-stage schizophrenia is associated with impaired working memory, but this may not be the case with other antipsychotics. Along the same lines, although working memory may be impaired, other cognitive functions may be improved by this same treatment. Thus, it is important to consider the type of cognitive function and the dose and duration of treatment before concluding that atypical antipsychotics will not be beneficial for cognitive outcomes.

This hope and aspiration appears to be what keeps atypical antipsychotics in the forefront of medications prescribed for the entire spectrum of psychosis. Prescribers continue to hope that there is merit in atypicals to enhance cognition. Furthermore, despite evidence that using other cognitive enhancers in conjunction with atypical anitpsychotics is effective in enhancing cognition (e.g. use of donepezil), this type of treatment is not frequently recommended.[36]

It is likely that it will take another major clinical trial to answer this question properly. In the meantime, one hopes that the outcomes of newer initiatives to find new molecules for improving cognition are successful in making a difference to our patients’ lives. Recent research has shown that treatment with minocycline has a beneficial effect on cognitive functioning, particularly in terms of executive functions like working memory, cognitive shifting, and cognitive planning.[35] Research initiative underway from National Institute of Mental Health USA (MATRICS) to find new targets for cognition enhancement and to develop new molecules is an important initiative.[37]

As the literature base supports the beneficial effects of acute nicotine administration on cognitive deficits in individuals with schizophrenia, the importance of introducing selective agents for these receptors is emphasized for determining their therapeutic roles. Additionally, using dopamine (D_1_) agonists represents an approach that may prove to be successful. Finally, glutaminergic agents are reported to be potentially useful in reversing and preventing neurocognitive dysfunction.[37] Hopefully, in the near future our patients will be able to expect better outcomes from schizophrenia and a better quality of life.

The significance of cognition in schizophrenia has now opened newer vistas for clinical practice. Psychiatrists in community as well as in tertiary care facilities will be expected to have facilities and skills for adequate measurements. The reliance upon cognitive factors for objectivity in diagnosis and treatment is likely to change the practice and prescribing. Together with advances in pharmacogenomics and the possibility of adequate prediction of effects and side effects, the prescribing practice is likely to become challenging and complex. We need to be ready with more education and facilities at hand to be able to provide the standard of care in communities.

Future research is expected to highlight mechanism of cognitive dysfunction that leads to deterioration in schizophrenia, provide reliable neurobiological explanation for a number of successful psychological therapeutic interventions and address the issue of cognitive enhancement in at-risk psychosis. The cognition-schizophrenia relationship has become more pronounced and rich. It is visible with much more clarity. Service users and consumers are looking at this area with hope and excitement not only for treatment of schizophrenia, but for prevention as well.
